# High-efficiency exfoliation of large-area mono-layer graphene oxide with controlled dimension

**DOI:** 10.1038/s41598-017-16649-y

**Published:** 2017-11-27

**Authors:** Won Kyu Park, Yeojoon Yoon, Young Hyun Song, Su Yeon Choi, Seungdu Kim, Youngjin Do, Junghyun Lee, Hyesung Park, Dae Ho Yoon, Woo Seok Yang

**Affiliations:** 1 School of Advanced Materials Science and Engineering, Sungkyunkwan University, 2066 Seobu-ro, Jangan-gu, Suwon-si, Gyeonggi-do 16419 Republic of Korea; 2 Electronic Materials and Device Research Center, Korea Electronics Technology Institute (KETI), 25 Saenari-ro, Bundang-gu, Seongnam-si, Gyeonggi-do 13509 Republic of Korea; 3 Department of Nanotechnology and Advanced Material Engineering, Sejong University, 209 Neungdong-ro, Gwangjin-gu, Seoul 05006 Republic of Korea; 4 Department of Materials Engineering, Korea Aerospace University, 76 Hanggongdaehak-ro, Deogyang-gu, Goyang-si, Gyeonggi-do 10540 Republic of Korea; 5 Department of Energy Engineering, School of Energy and Chemical Engineering, Low Dimensional Carbon Materials Center, Perovtronic Research Center, Ulsan National Institute of Science and Technology (UNIST), Ulsan, 44919 Republic of Korea

## Abstract

In this work, we introduce a novel and facile method of exfoliating large-area, single-layer graphene oxide using a shearing stress. The shearing stress reactor consists of two concentric cylinders, where the inner cylinder rotates at controlled speed while the outer cylinder is kept stationary. We found that the formation of Taylor vortex flow with shearing stress can effectively exfoliate the graphite oxide, resulting in large-area single- or few-layer graphene oxide (GO) platelets with high yields (>90%) within 60 min of reaction time. Moreover, the lateral size of exfoliated GO sheets was readily tunable by simply controlling the rotational speed of the reactor and reaction time. Our approach for high-efficiency exfoliation of GO with controlled dimension may find its utility in numerous industrial applications including energy storage, conducting composite, electronic device, and supporting frameworks of catalyst.

## Introduction

Graphene, *sp* ^2^-hybridized carbon structure with two-dimensional atomic layer, possesses remarkable physical and chemical properties, and has drawn worldwide research efforts since its first experimental demonstration, such as micro- or nano-electronics and energy storage or harvesting^1–8^. The material properties are largely affected by its specific atomic structures and morphologies. In particular, the lateral size of graphene plays a significant role in electrical and thermal conductivities: e.g., large-area graphene can be utilized as the transparent conductive film^9–11^. Therefore, developing a synthesis method with controllable geometrical morphologies and dimension, and also with large-scale production capability, is crucial for the widespread industrial application of graphene.

Among various synthesis routes developed so far, chemical vapor deposition (CVD) technique is known to produce large-area graphene sheets with desirable qualities^12–16^. However, current CVD technology is limited in high-yield manufacturing processes due to the its multifaceted synthesis and transfer steps and associated costs. Alternatively, chemical methods involving oxidation, reduction, and exfoliation processes using bulk graphite have been intensively studied as an effective and economical approach toward the manufacturing of graphene due to its facile solution processability^9,17–29^. In solution-based chemical processes, controlling the resulting flake size is vital since the lateral size of the flake has direct impact on the overall physical properties^17,25,28,30^.

Currently, graphene oxide (GO) is one of the most widely studied materials synthesized by the solution processed chemical approach^9,11,17–32^. However, making GO with large lateral size is challenging because the flakes are easily torn from the sonication treatment during the exfoliation process of graphite oxide (GtO). For this reason, typical size of GO and reduced graphene oxide (rGO) flakes ranges from hundred nanometers to a few microns in lateral dimension^18,33–36^, and there have been many efforts to overcome this issue. Tung *et al*. reported GO flakes with lateral size of ~40 µm through versatile solution-based process^37^. Zhao *et al*. reported GO flakes with lateral dimension of ~100 µm by modifying the oxidation conditions from the Hummers’ method^30^. However, the yield of single-layer GO was relatively low (~10%) in both cases. Luo *et al*. reported single-layer GO with high yield of ~90% by microwave-assisted chemical method, but the flake dimension was only ~200 µm^2^ in area^11^. Furthermore, microwave-assisted chemical method typically requires long synthesis time and complicated oxidation processes.

Herein, we report a facile method to exfoliate GtO into large-area and single- or few-layer GO with high yield using shearing stress. The shearing stress reactor consists of two coaxial cylinders with the inner one rotating. Toroidal vortices, regularly spaced along the cylinder axis, are generated at critical rotating speed, which creates shearing stress to the reactants placed between the cylinders^38–40^. We found that this toroidal motion of fluids leads to highly efficient exfoliation to the lateral direction without damaging the GtO. Moreover, lateral size of the exfoliated GO could be readily tuned by controlling the rotational speed of the inner cylinder. To confirm the efficacy of the proposed approach, well-known exfoliation techniques, sonication and homogenization, were comparatively evaluated along with the shearing stress method.

## Results and Discussion

GtO is typically fabricated by the inter-layer oxidization of natural graphite with oxidizing agents such as KMnO_4_^41–43^. As synthesized GtO contains carbonyl groups on the edge sites and hydroxyl and epoxy groups in the basal plane, which expand the inter-layer spacing of the natural graphite^17,21^. GtO, with its inter-layer space being expanded, can then be readily exfoliated into GO with single- or multi-layers due to the weakened inter-layer van der Waals forces^18,23^.

**Figure 1:**
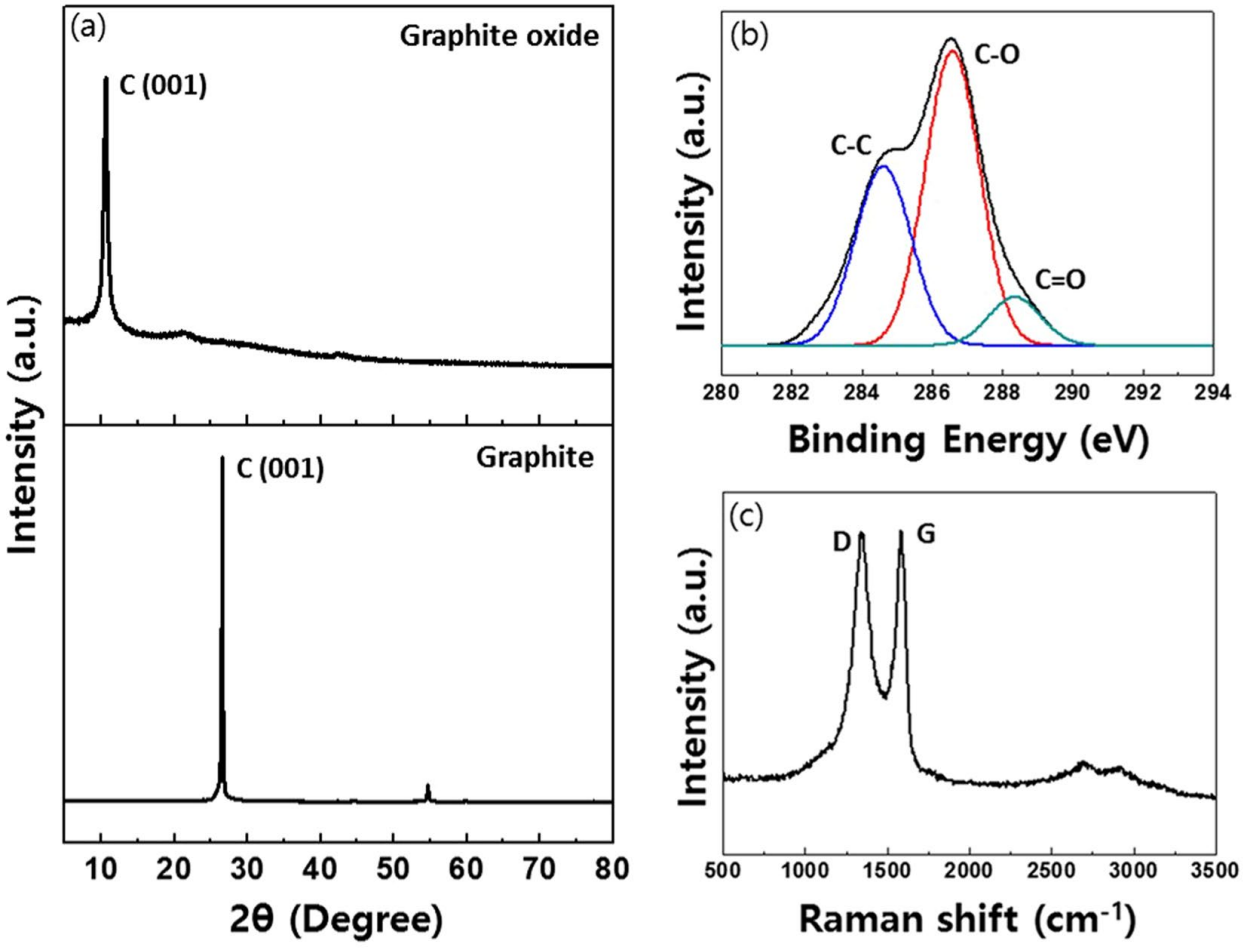
Spectroscopic analysis of GtO produced by shearing stress reactor. (**a**) XRD pattern of GtO at 10.3° (d-spacing: ~8.5 Å, upper) and graphite powder at 26.5° (d-spacing: ~3.4 Å, lower). (**b**) C1s XPS spectra of C-C bond at 284.5 eV (blue), C–O bond at 286.7 eV (red), and C=O bond at 288.2 eV (green). (**c**) Raman spectra of GtO with D (1349 cm^−1^) and G peak (1589 cm^−1^). 514 nm excitation.

GtO produced via the oxidation reaction (60 min) in shearing stress reactor was spectroscopically analyzed as shown in Fig. 1 ^44^. Fig. 1a shows the x-ray diffraction (XRD) spectra of the produced GtO powders where the main peak of graphite at 26.5° (lower) is reduced to 10.3° after the oxidation reaction (upper). The inter-planar spacing of GtO (0.85 nm), measured by Bragg’s Law ($n\lambda =2{dsin}\theta$), was larger than that of the natural graphite (0.34 nm) due to the oxygen functional groups present on the carbon sheet^17,21^. Figure 1b shows the x-ray photoelectron spectroscopy (XPS) C1s spectra of GtO. Three main peaks from different functional groups of carbon atoms are clearly observed: carbon bond (C–C) at 284.5 eV, epoxy/hydroxyls group (C–O) at 286.7 eV, and carbonyl group (C=O) at 288.2 eV. Typical Raman spectra of graphene have two distinctive peaks at 1590 cm^−1^ and 1350 cm^−1^, G mode representing the *sp* ^2^ C element and D mode representing the degree of defects in the *sp* ^2^ domain, respectively. As expected and shown in Fig. 1c, Raman spectra of GtO exhibit G band at 1589 cm^−1^ and pronounced D band at 1349 cm^−1^ originating from the reduced plane size of *sp* ^2^ domains owing to the intensive oxidation.

**Figure 2:**
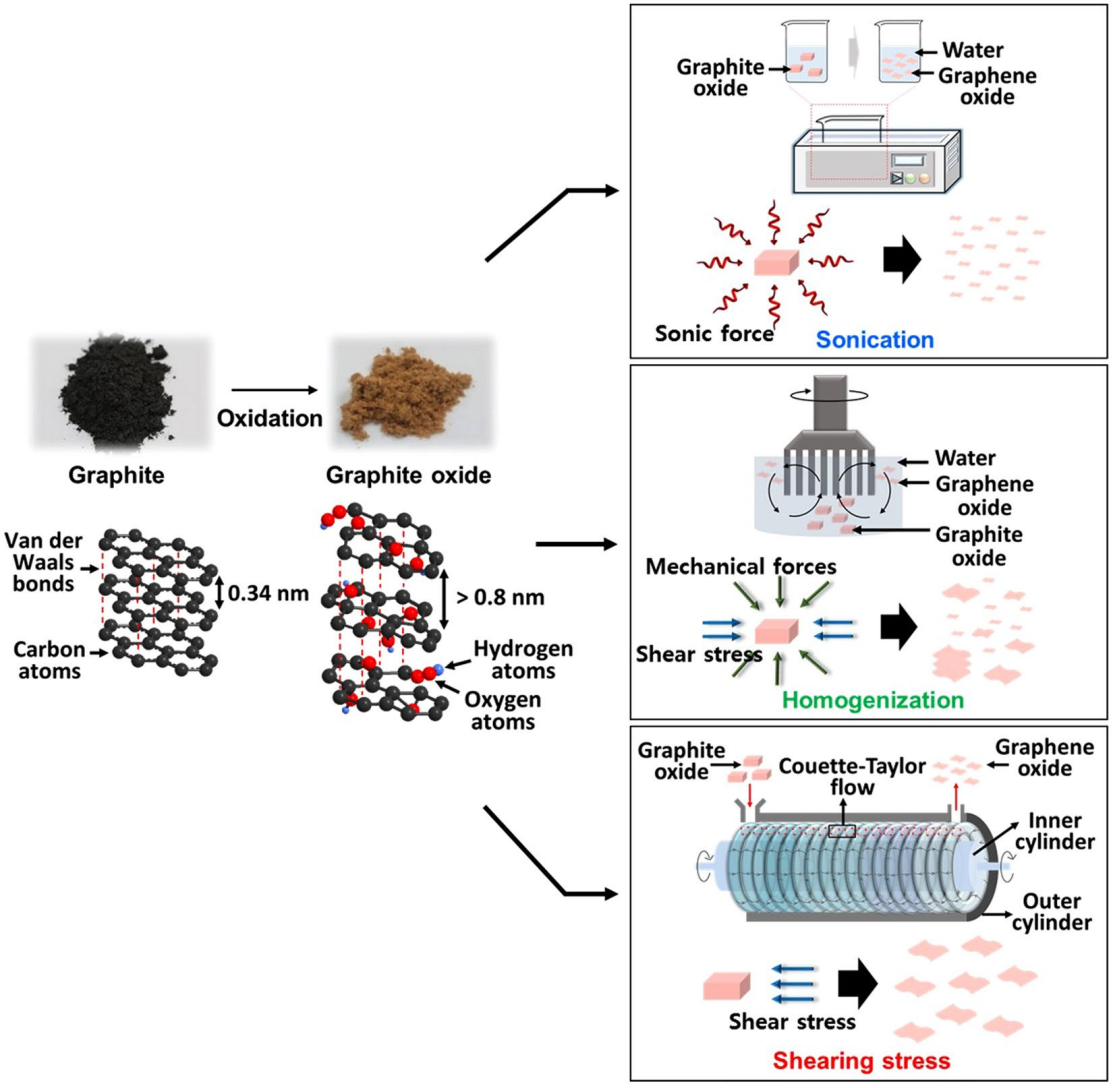
Schematic of various exfoliation methods of GtO (sonication, homogenization, and shearing stress).

**Figure 3:**
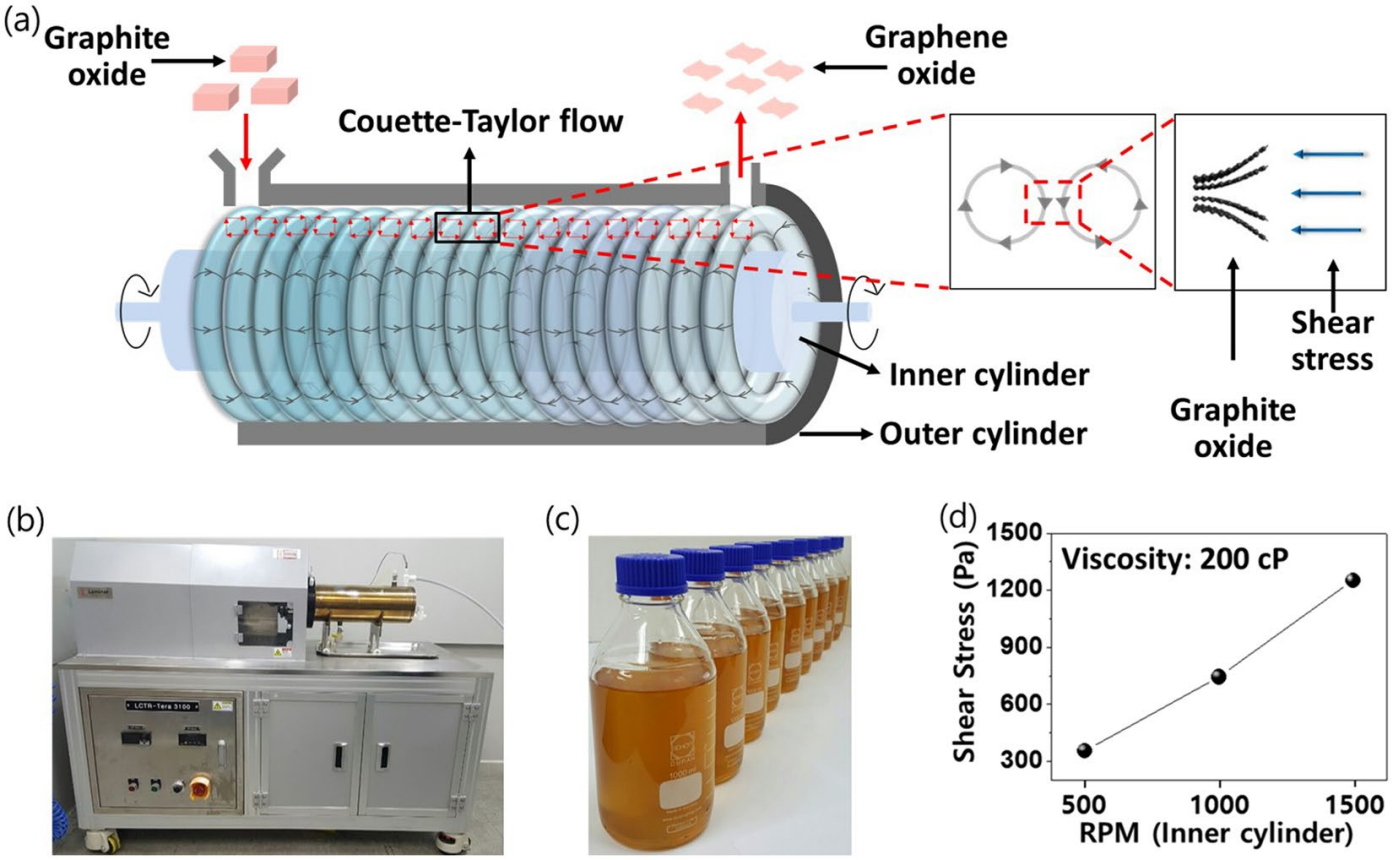
Production of GO via shearing stress reactor. (**a**) Illustration of the exfoliation process of GtO flakes and conceptual diagram of vortex structure inside the shearing stress reactor. (**b**) Photograph of shearing stress reactor. (**c**) GO-water dispersions produced by shearing stress reactor. (**d**) Shear stress of fluid in the reactor with varying rotational speeds of the inner cylinder.

Figure 2 illustrates three different approaches (sonication-upper, homogenization-mid, and shearing stress-lower) for the GtO exfoliation process studied in this work. The GtO was prepared under the same experimental conditions and the exfoliation time was kept constant for all cases. Sonication is the most widely used approach for the exfoliation process and the single-layered GO flakes can be readily synthesized by the ultrasonic force (directionless force)^18,33,34,45^. In the homogenization, exfoliation takes place from the lateral side of GtO by the shearing stress and mechanical force, which is known to produce single-layered GO with relatively large lateral size^45^. The shearing stress reactor comprises two coaxial cylinders. While the outer cylinder remains standstill, the inner one rotates at controlled speed. When the rotational speed of the inner cylinder reaches a threshold value, doughnut-shaped vortexes are generated which rotates in opposite directions with constant arrays along the cylinder axis^38–40^. This shearing stress flow induces highly effective radial mixing and uniform fluidic motion within each vortex cell, enabling enhanced mass transfer of the reactants. The toroidal motion also generates high wall shear stress, which can facilitate the GtO exfoliation (Fig. 3a).

In shearing stress reactor, the hydrodynamic condition of the fluids depends on the rotating speed of the inner cylinder. The shearing stress flow is formed when the Taylor number (Ta) proportional to the angular velocity of the inner cylinder exceeds a critical value, which is determined by the following relation^38–40^:

1 $${\rm{Ta}}={(\frac{d}{{R}_{1}})}^{1/2}\frac{{\omega }_{1}{R}_{1}d}{\nu }$$

where *R*_1_ is the radius of inner cylinder, *ω*_1_ is the angular velocity of the inner cylinder, *d* is the width of the annular gap, and *ν* is the kinematic viscosity. In this work, the viscosity was set at 200 cP (water-dispersed GtO used for forming the shearing stress flow) and the exfoliation was carried out from 500 to 1500 rpm for 1, 3, and 5 hr.

Figure 3b,c show the shearing stress reactor and its capability of large-volume GO production. The generated shearing stress, dependent on the speed of rotating inner cylinder, is plotted in Fig. 3d: 355.0 Pa at 500 rpm, 744.2 Pa at 1000 rpm, and 1250.8 Pa at 1500 rpm, respectively. As expected, shearing stress increases with rotational speed, indicating more efficient GtO exfoliation at higher shear force.

The efficacy of various exfoliation methods was evaluated by analyzing the aggregated non-exfoliated GtO particulates. GtO was exfoliated in water, and the exfoliated GO and non-exfoliated GtO were isolated by centrifugation (500 rpm) after the exfoliation process. During centrifugation, non-exfoliated GtO particulates were precipitated out, while exfoliated GO remained in the supernatant. We note that the exfoliated GO in the supernatant was mostly single- or few-layer, and its measured weight relative to that of the initial GtO was used to determine the recovery rate.

**Figure 4:**
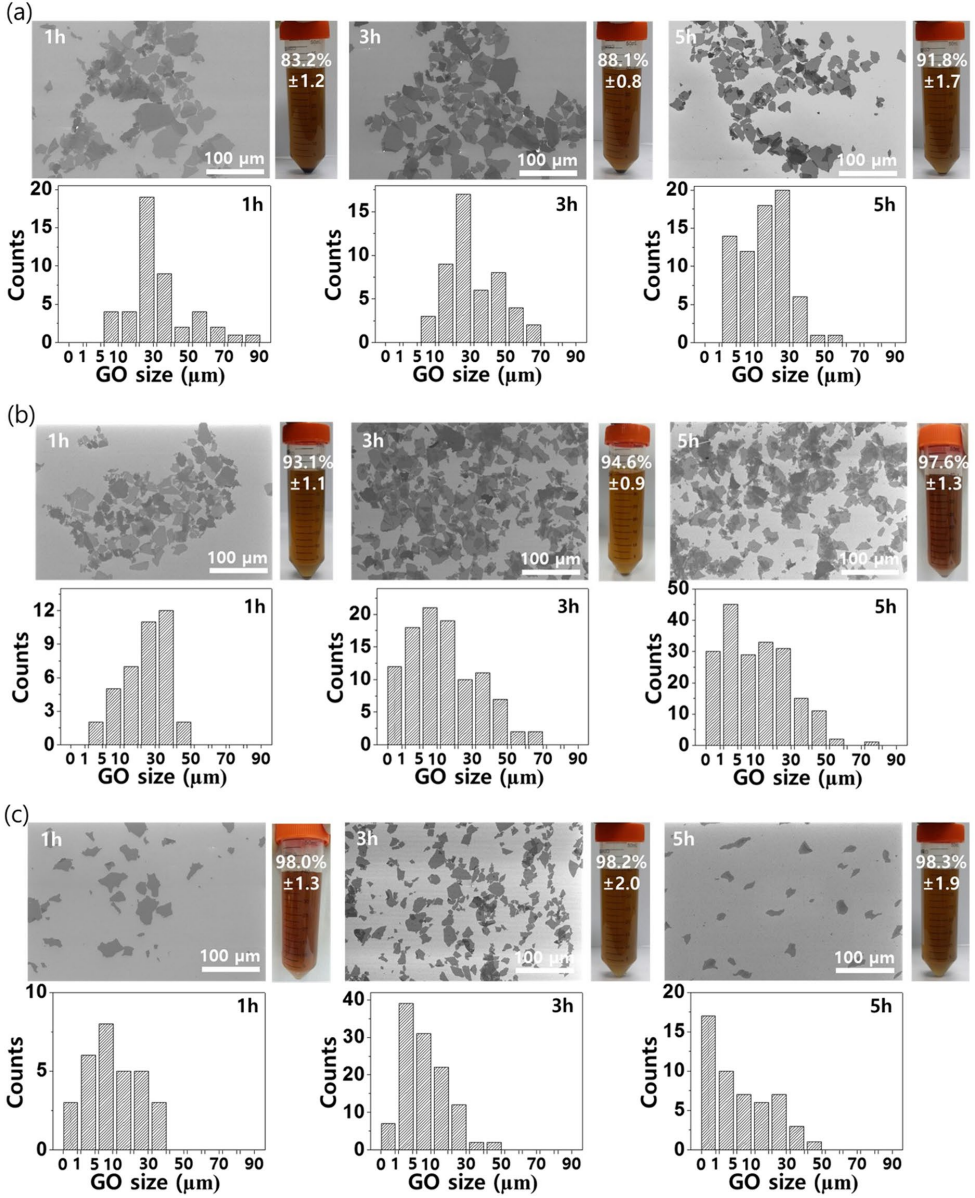
FE-SEM images and recovery rates of the exfoliated GO produced by shearing stress reactor: Inner cylinder rotating speed of (**a**) 500 rpm, (**b**) 1000 rpm, and (**c**) 1500 rpm with exfoliation time of 1, 3, and 5 hr, respectively. Flake size distributions from each condition is provided in the histogram. Photograph: GO dispersion in water after the centrifugation. Precipitates indicate the non-exfoliated GtO flakes.

Field emission scanning electron microscopy (FE-SEM) images and recovery rates of exfoliated GO obtained from the shearing stress are shown in Fig. 4. The exfoliation time was varied from 1 to 5 hr, while the rotating speed of inner cylinder was set as 500, 1000, and 1500 rpm. At 500 rpm (Fig. 4a), the lateral size of exfoliated GO was ~70, ~50, and ~30 µm and the recovery rate was 83.2, 88.1 and 91.8%, at each time duration. At 1000 rpm, the recovery rate increased over 93% even at 1 hr of exfoliation time, and the overall flake size was ~50 µm (Fig. 4b). At 1500 rpm, the GO flake size ranged 10 ~ 30 µm, and the recovery rate was over 98% at all exfoliation times, indicating that most of GtO was successfully exfoliated and well-dispersed in water (Fig. 4c). Figure S1 shows the UV-vis spectrum according to the recovery rates of GO dispersed in water. GO only absorbs in the violet and UV range of light and has a maximum absorption at 231 nm. As the yield of single- or few-layer GO increased, the concentration was increased and the absorption rate increased accordingly.

Table 1 shows the lateral dimension and recovery rate of exfoliated GO via sonication and homogenization method under various experimental conditions (Supplementary Figures S2, 3). For the sonication method at 80 W of sonication power, the lateral size of GO was large in general: ~50, ~40, and ~20 µm with sonication time of 1, 3, and 5 hr, respectively. However, the recovery rate was only 16.2% even at 5 hr of sonication time, indicating that most of the GtO precipitated without further exfoliation. Under this mild-sonication condition, large-size GO can be obtained, but achieving single-layer GO with high-yield is limited since the exfoliation cannot fully occur. At higher sonication power of 150 W, the exfoliation process was almost completed (recovery rates were 92.1, 98.3, and 98.9% at 1, 3, and 5 hr, respectively) but with decreased flake size (~0.5 to 5 µm range) and nonuniform flake distributions. With the sonication power further increased at 200 W, the exfoliation of GtO almost fully occurred: the lateral size and recovery rates were ~0.8, ~0.5, and ~0.3 µm, and 98.8, 99.0 and 99.1% at 1, 3, and 5 hr, respectively. As shown here, sonication method is intrinsically limited in producing single-layered GO flakes with large lateral size since the sonication generates acoustic wave agitation which can easily damage the starting GtO.

**Table 1:** Lateral dimension and recovery rate of exfoliated GO flakes via sonication and homogenization method under various experimental conditions.

Exfoliation method	Time (h)	Condition	Lateral dimension (µm)	Recovery rate (%)
Sonication	1	80 W	~50	7.3 ± 0.7
	3	80 W	~40	11.7 ± 0.6
	5	80 W	~20	16.2 ± 0.8
	1	150 W	~5	92.1 ± 1.9
	3	150 W	~5	98.3 ± 2.0
	5	150 W	~0.5	98.9 ± 2.1
	1	200 W	~0.8	98.8 ± 1.3
	3	200 W	~0.5	99.0 ± 1.9
	5	200 W	~0.3	99.1 ± 2.9
Homogenization	1	3000 rpm	~40	6.1 ± 0.8
	3	3000 rpm	~30	13.5 ± 0.5
	5	3000 rpm	~15	26.7 ± 1.6
	1	6000 rpm	~25	21.3 ± 0.4
	3	6000 rpm	~15	24.3 ± 1.6
	5	6000 rpm	~10	32.2 ± 1.0
	1	9000 rpm	~20	49.1 ± 1.9
	3	9000 rpm	~15	50.6 ± 1.2
	5	9000 rpm	~10	71.2 ± 2.2

Sonication power: 80, 150, and 200 W. Homogenization rotation speed: 3000, 6000, and 9000 rpm. Exfoliation time: 1, 3, and 5 hr.

For the homogenization method at 3000 rpm rotational speed, the lateral size of exfoliated GO were ~40, ~30, and ~15 µm with homogenization time of 1, 3, and 5 hr, respectively, and the corresponding recovery rates were only 6.1, 13.5, and 26.7%, leaving most of the GtO unexfoliated. At 6000 rpm, the flake size was ~25 µm or less at all exfoliation time but the recovery rate was still 32.2% even at 5 hr of homogenization time. The recovery rate was somewhat improved at 9000 rpm (71.2% at 5 hr), but the corresponding flake size was less than 10 µm. Overall, the homogenization process results in low recovery rates with nonuniform size distributions, and the frictional force generated between the inner and outer blades during the rotation causes significant damages to the exfoliated GO flakes. Consequently, the yield of large-area and single-layer GO is also quite low in this method.

Based on these results, we conclude that sonication method typically produces GO flakes with small sizes due to the destructive acoustical wave agitation in the solution, and homogenization method results in GO with various flake size distributions because the GO sheets are readily destroyed by the mechanical forces of the blade. On the other hand, the shearing stress process promotes the non-destructive exfoliation of GtO owing to the slippage of the GO induced by the in-plane directed shear stress, which is described as the rheologically derived GO.

**Figure 5:**
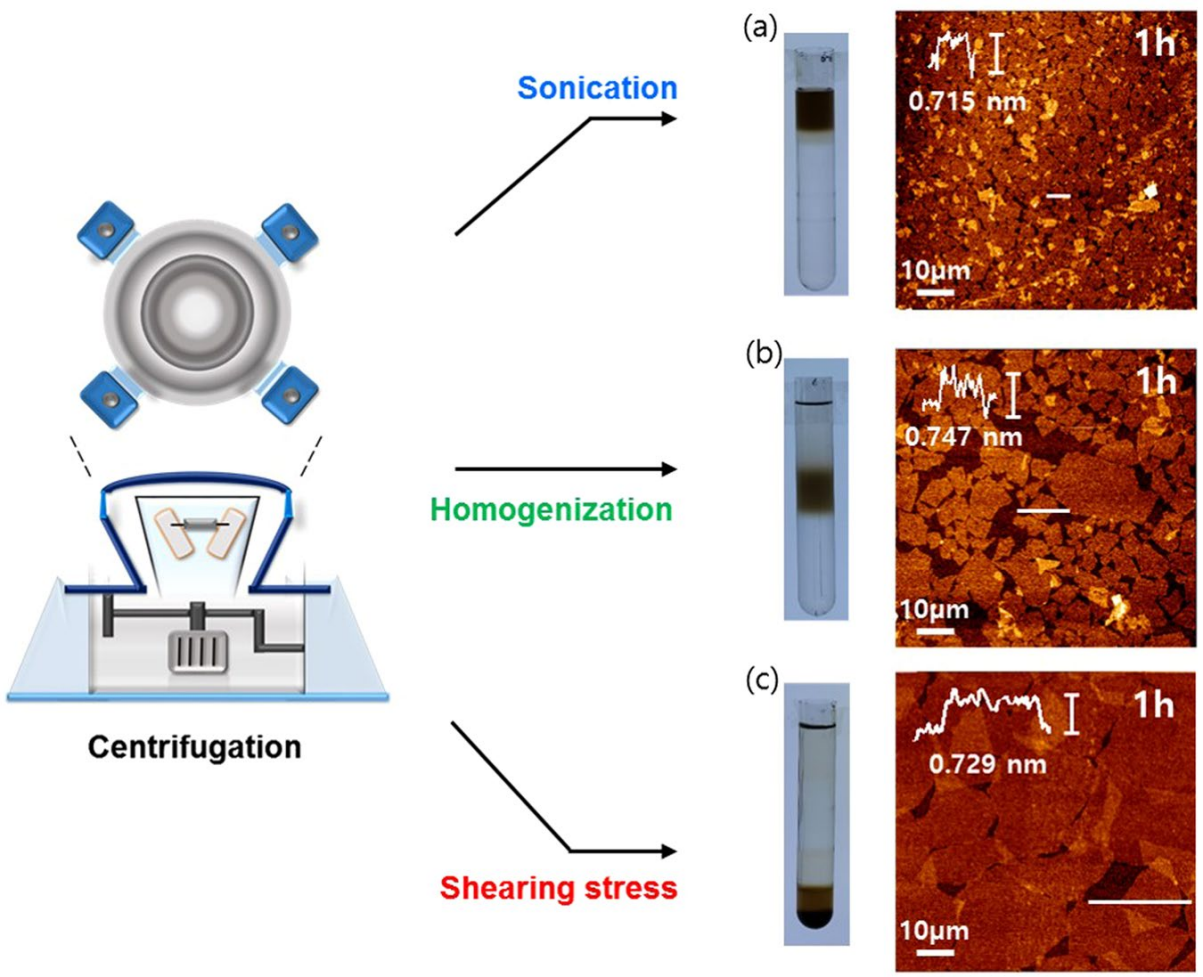
Tapping-mode AFM images and height profiles of GO sheets. Comparison of the lateral size and thickness of exfoliated GO prepared by (**a**) sonication, (**b**) homogenization, and (**c**) shearing stress reactor. Photograph: GO dispersion in water after the centrifugation at 6000 rpm for 30 min.

Atomic force microscopy (AFM) was performed to analyze the thickness of GO flakes prepared by three exfoliation methods - sonication, homogenization, and shearing stress - for exfoliation time of 1 hr and after centrifuging at 6000 rpm for 30 min (Fig. 5). The height profiles reveal uniform flake thickness of ~0.75 nm, typical of the exfoliated individual GO sheet, whereas the lateral size exhibited distinctive size variations: ~5, ~20, and ~50 µm range for sonication (150 W), homogenization (6000 rpm), and shearing stress (1000 rpm), respectively. This difference in size distribution positioned the dispersed exfoliated flakes at different locations of the conical tube (upper, mid, and lower region) due to varying flake weights as shown in the digital image of Fig. 5.

Compared to the conventional sonication and homogenization method, the recovery rate of shearing stress improved more than 90% with associated high-yield of single- or few-layered GO with large lateral size. The strong shear stress generated from the vortex in the reactor also enabled non-destructive exfoliation. These results suggest that shearing stress approach can afford effective exfoliation of GtO flakes with desired size tunability and quality, realizing facile production of single- or few-layer GO in high yields.

## Conclusions

In summary, we have demonstrated a facile method to promote the non-destructive exfoliation process of GtO into GO using shearing stress reactor. We found that the shear stress generated inside the reactor enabled efficient exfoliation of GtO with tunable size and high yield of single- or few-layer GO sheets. In this work, large-area single-layer GO flakes over 80% yield was obtained, surpassing the conventional sonication or homogenization method. Our approach for facile and large-scale production of GO thus has great potentials in various industrial applications including energy storage and harvesting, conducting composites, or electronic devices.

## Materials and Methods

### Preparation of GtO

Graphite flakes (150 µm, Alfa Aesar) were oxidized using the shearing stress reactor (Supplementary Figure S4, Lamina co. ltd). 7 g of graphite flakes was added to 250 mL of sulfuric acid (H_2_SO_4_, 95%, Sigma Aldrich). Then, 32 g of potassium permanganate (KMnO_4_, Sigma Aldrich) was slowly added to the mixture at ~10 °C, and stirred for 30 min. Shearing stress reactor (length: 500 mm) consists of two coaxial cylinders with the fixed outer cylinder (radius: 68 mm) and the rotating inner cylinder (radius: 60 mm). After the mixture was introduced into the gap between the two cylinders, the inner cylinder was rotated. Oxidation of graphite inside the shearing stress reactor led to brown-colored slurry. Then, 250 mL of purified water and 15 mL of hydrogen peroxide (H_2_O_2_, 30%, Sigma Aldrich) were added to the mixture, and stirred for 30 minutes. For purification, centrifugation was used to separate the GtO from the impurities. Finally, the dried GtO powders were obtained by freeze-drying.

### Exfoliation of GtO for GO

In this study, three different exfoliation methods were carried out to prepare GO from GtO. All exfoliation methods were conducted for 1, 3, and 5 hr duration. Variable parameters were power (80, 150, and 200 W) for the sonication method, rotating speed of axis (3000, 6000, and 9000 rpm) for the homogenization method, and rotating speed of inner cylinder (500, 1000, 1500 rpm) for the shearing stress reactor, respectively. In the case of sonication and homogenization method, GtO was dispersed in water with concentration of 1 mg/mL, whereas the viscosity of GtO dispersed in water was set at 200 cP for the shearing stress reactor. Non-exfoliated GtO particles were precipitated out by centrifugation at 500 rpm for 10 min. The supernatant containing single- or few-layer GO was obtained and coated on Si substrate for further characterization.

### Characterization

The microstructure and lateral size of the samples was investigated using field emission scanning electron microscopy (JSM-7600F, JEOL). The thickness and lateral size of GO sheets were obtained using atomic force microscopy (SPA-300HV, SII). X-ray diffraction patterns of oxidized graphite were determined by D8 ADVANCE (Bruker Corporation) with Cu-Ka X-ray source. Raman spectra were obtained by micro-Raman system (Bruker FRA 160/S, Bruker) with excitation energy of 2.41 eV. X-ray photoelectron spectroscopy spectra of GO samples were obtained by VG Microtech ESCA 2000 (JEOL) with a monochromatic Al-Ka X-ray source at 250 W. The UV-Vis absorption spectrum was obtained by using a TIDAS 100 spectrophotometer (J&M, Germany). The spectrum has been recorded by measuring a 0.02 wt% solution of GO dissolved in water.

## Electronic supplementary material

Supplementary Information
