# Supplementary material for: High-efficiency exfoliation of large-area mono-layer graphene oxide with controlled dimension
Source: Sci Rep. 2017 Nov 27;7:16414. doi: 10.1038/s41598-017-16649-y (PMC5704010; doi:10.1038/s41598-017-16649-y)
Supplement: Supplementary file 1 — Supplementary Information [file 41598_2017_16649_MOESM1_ESM.doc]

**Supporting Information**

**High-efficiency exfoliation of large-area mono-layer graphene oxide with controlled dimension**

Won Kyu Park ^1†^, Yeojoon Yoon ^2†^, Young Hyun Song ^3†^, Su Yeon Choi ^2^, Seungdu Kim ^2,4^, Youngjin Do ^2^, Junghyun Lee ^5^, Hyesung Park ^5*^, Dae Ho Yoon ^1*^ and Woo Seok Yang ^2*^

^1^ School of Advanced Materials Science and Engineering, Sungkyunkwan University, 2066 Seobu-ro, Jangan-gu, Suwon-si, Gyeonggi-do, 16419, Republic of Korea.

^2^ Electronic Materials and Device Research Center, Korea Electronics Technology Institute (KETI), 25 Saenari-ro, Bundang-gu, Seongnam-si, Gyeonggi-do, 13509, Republic of Korea.

^3^ Department of Nanotechnology and Advanced Material Engineering, Sejong University, 209 Neungdong-ro, Gwangjin-gu, Seoul 05006, Republic of Korea.

^4^ Department of Materials Engineering, Korea Aerospace University, 76 Hanggongdaehak-ro, Deogyang-gu, Goyang-si, Gyeonggi-do, 10540, Republic of Korea.

^5^ Department of Energy Engineering, School of Energy and Chemical Engineering, Low Dimensional Carbon Materials Center, Perovtronic Research Center, Ulsan National Institute of Science and Technology (UNIST), Ulsan, 44919, Republic of Korea.

* Address correspondence to hspark@unist.ac.kr (H. Park), dhyoon@skku.edu (D.H. Yoon), and wsyang@keti.re.kr (W.S. Yang).

^†^ These authors equally contributed to this work.


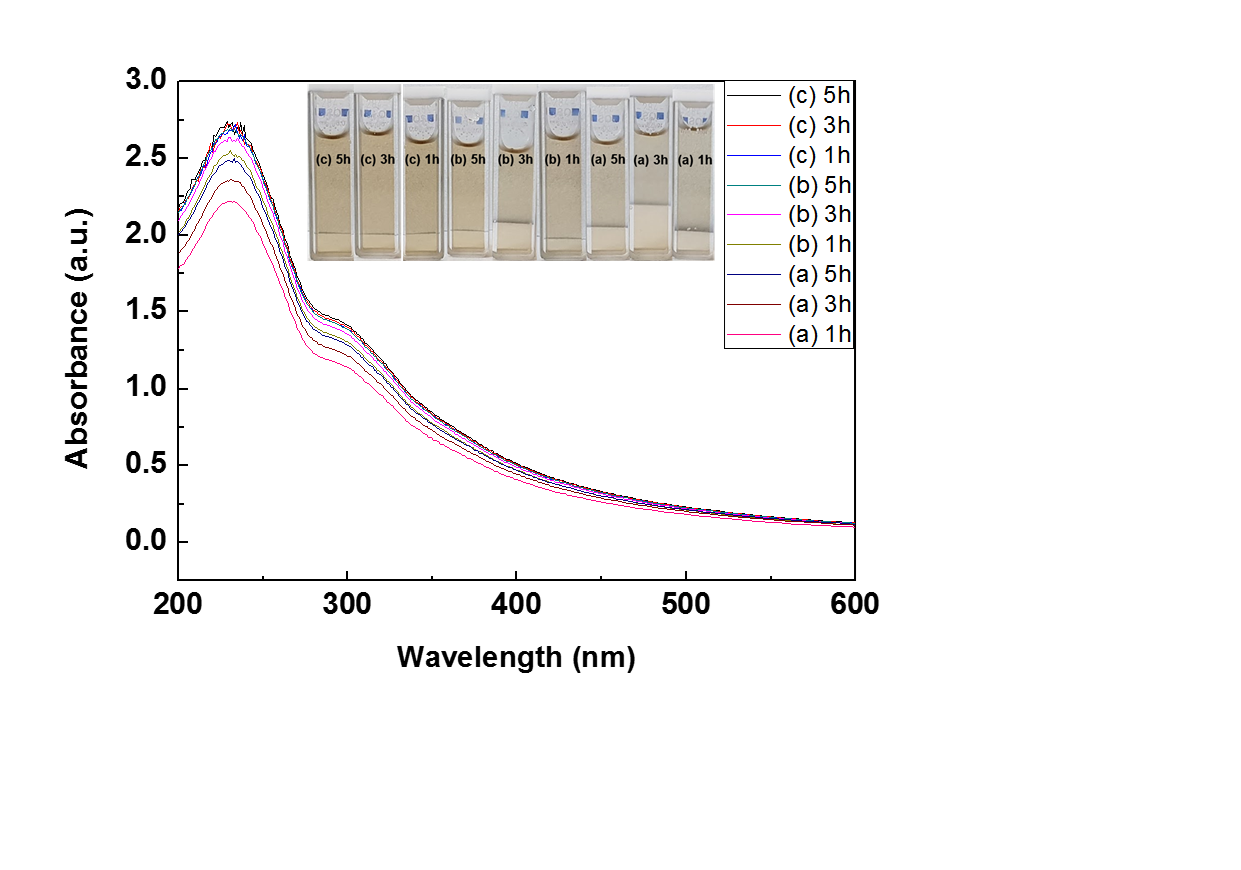


Figure S1. UV-vis spectrum according to the recovery rates of GO dispersed in water confirming the yield of monolayer GO after exfoliation by shearing stress (Inner cylinder rotating speed of (a) 500 rpm, (b) 1000 rpm, and (c) 1500 rpm with exfoliation time of 1, 3, and 5 hr, respectively). The inset shows the digital photographs of aqueous solution of GO.


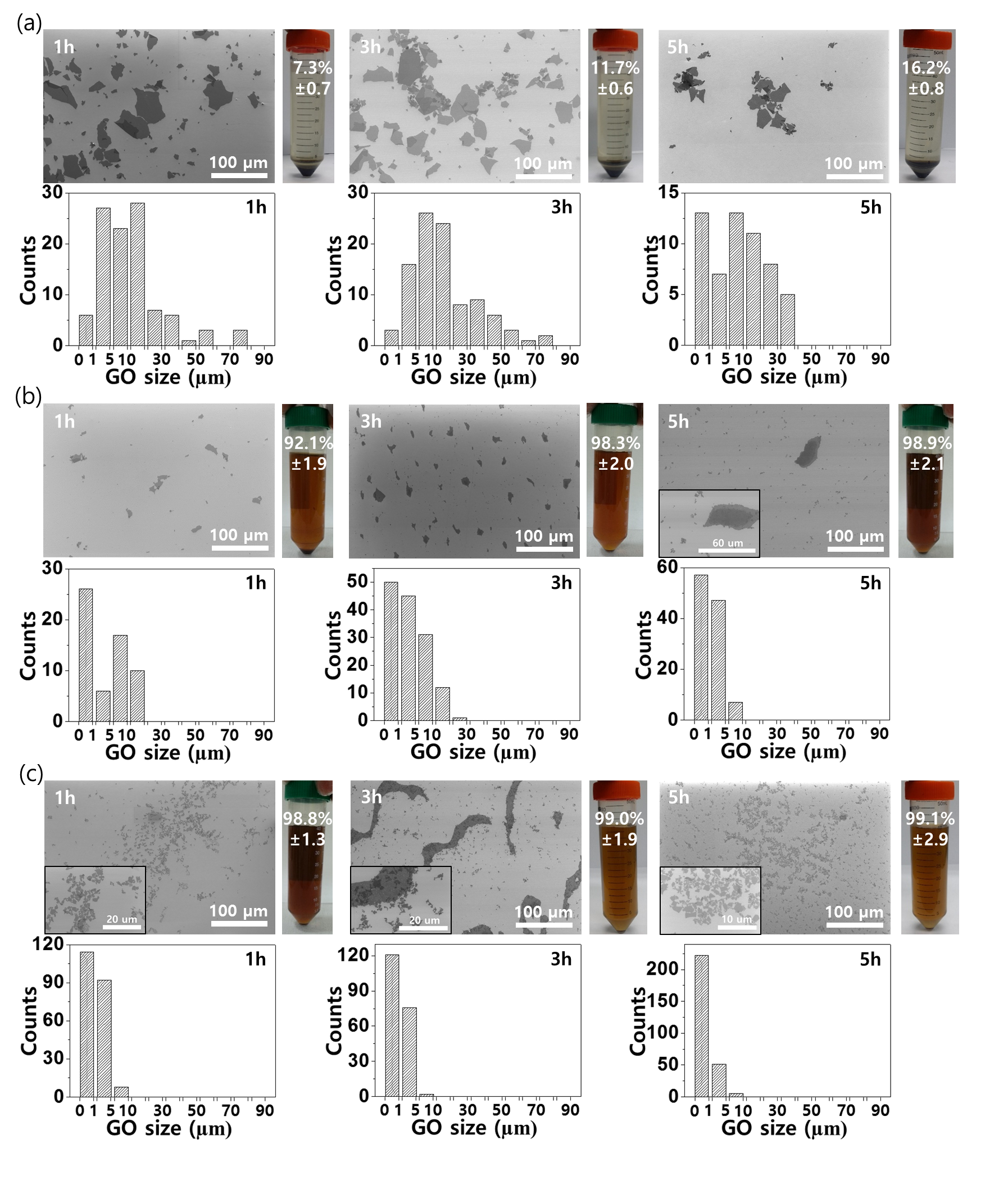


Figure S2. FE-SEM images and recovery rates of the exfoliated GO produced by sonication with varying power ((a) 80, (b) 150, and (c) 200 W) and time (1, 3, and 5 hr). Flake size distributions from each condition is provided in the histogram. Photograph: GO dispersion in water after the centrifugation. Precipitates indicate the non-exfoliated GtO flakes.


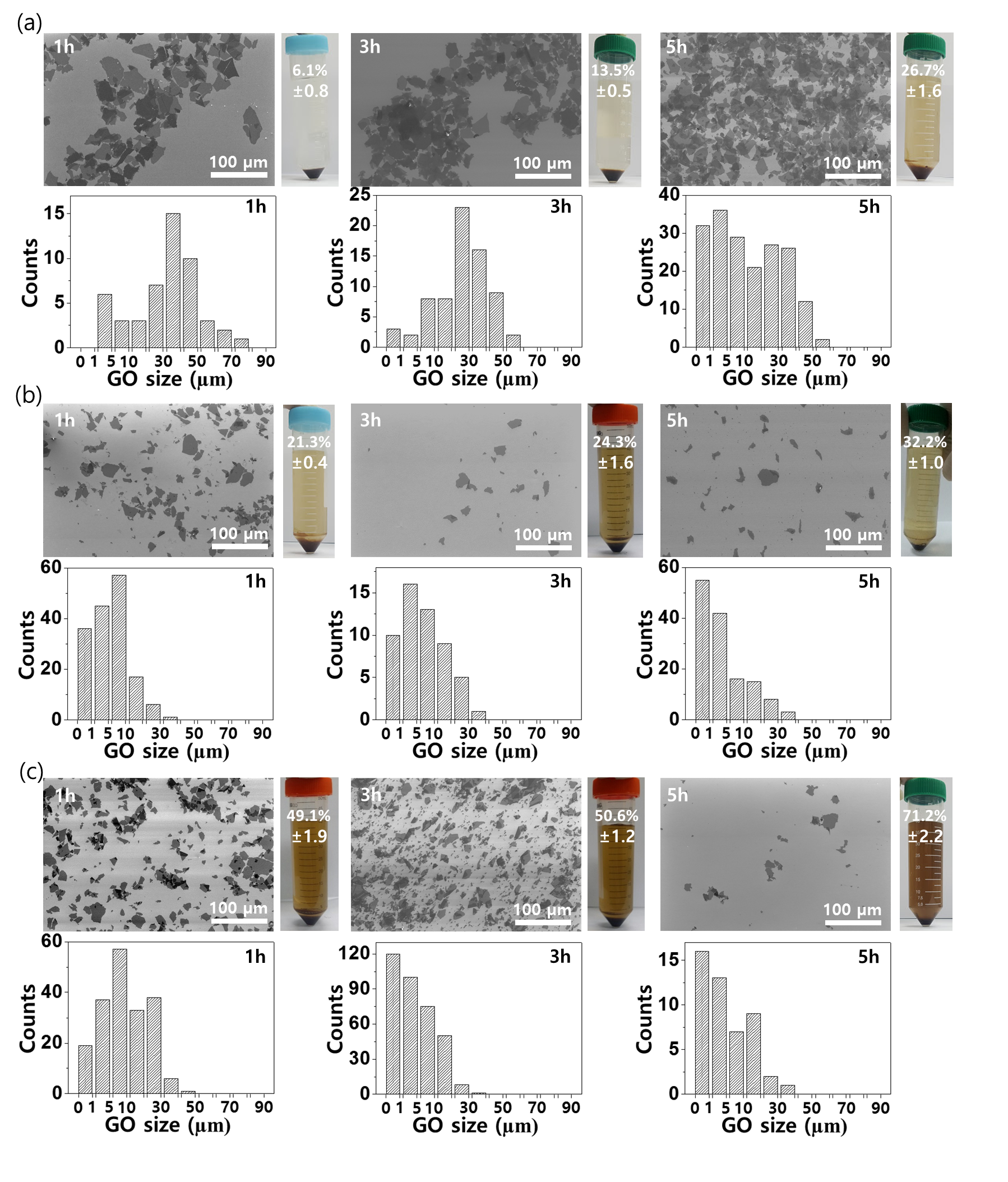


Figure S3. FE-SEM images and recovery rates of the exfoliated GO produced by homogenization with different rotating speed ((a) 3000, (b) 6000, and (c) 9000 rpm) and time (1, 3 and 5 hr). Flake size distributions from each condition is provided in the histogram. Photograph: GO dispersion in water after the centrifugation. Precipitates indicate the non-exfoliated GtO flakes.


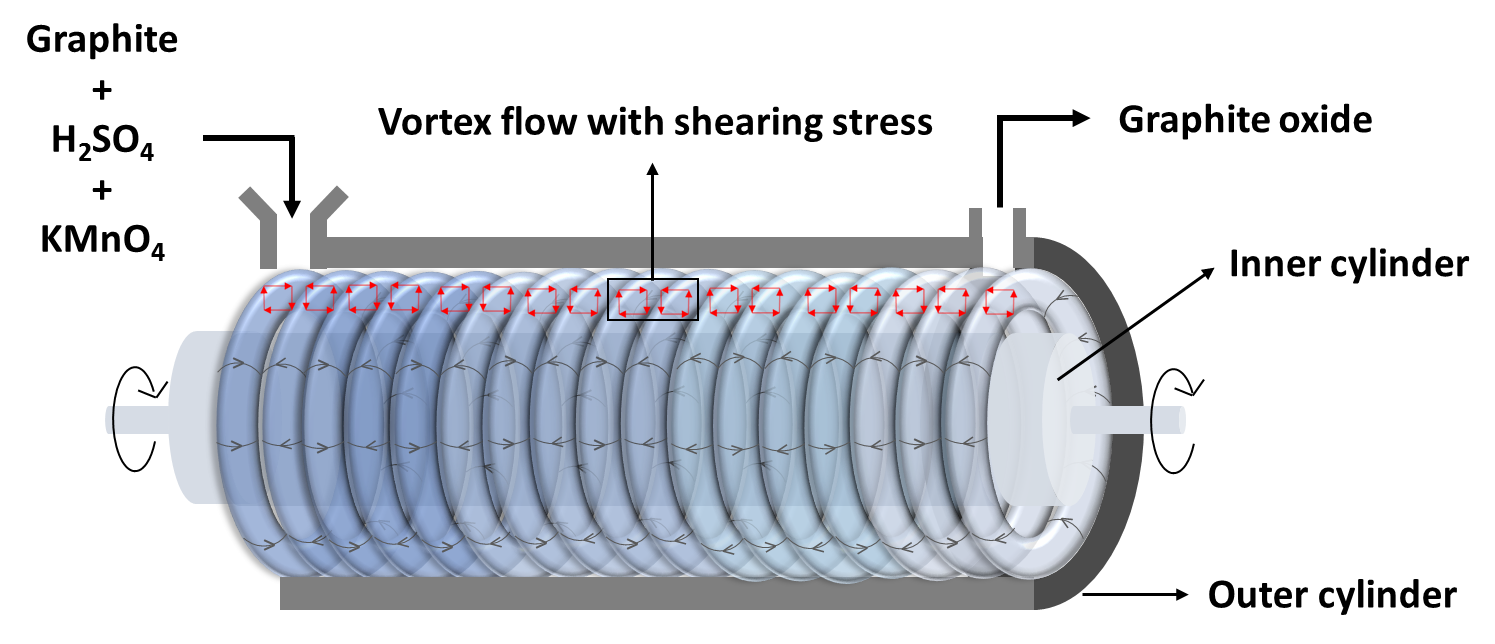


Figure S4. Illustration of the oxidation reaction process of graphite flakes and vortex structure generated inside the shearing stress reactor.
